# Changes in Frailty Status and Functional Capacity Following a Community-Based Nursing-Led Intervention in Older Women: An Exploratory Uncontrolled Pre–Post Study

**DOI:** 10.3390/healthcare14183060

**Published:** 2026-09-17

**Authors:** Candela Ortiz-García, Belinda Basilio-Fernández, Sonia Hidalgo-Ruíz, María del Pilar Alfageme-García, María Victoria Cáceres-Madrid, Manuel Martí Antonio

**Affiliations:** 1 Emergency Department, Clínica Zorrotzaurre, 48014 Bilbao, Spain; candelaorgar@gmail.com; 2 GICISA Research Group, Faculty of Nursing and Occupational Therapy, University of Extremadura, 10600 Plasencia, Spain; kirosony@unex.es (S.H.-R.); palfagemeg@unex.es (M.d.P.A.-G.); 3 Faculty of Podiatry, University of Extremadura, 10600 Plasencia, Spain; pgvicky@unex.es; 4 Department of Mathematics, University of Extremadura, 10600 Plasencia, Spain; mmartia@unex.es

**Keywords:** community-dwelling older adults, frailty, functional capacity, healthy ageing, nursing intervention, primary care

## Abstract

**Highlights:**

**What are the main findings?**

- The proportion of participants classified as frail according to the FRAIL Scale decreased from 47.8% at baseline to 8.7% at post-intervention assessment.
- Nine of eleven participants classified as frail at baseline moved to a less severe frailty category at follow-up.

**What is the implication of the main finding?**

- The observed pre–post changes support further evaluation of community-based nursing programmes in larger controlled studies.

**Abstract:**

**Background/Objectives:** Frailty is a common geriatric syndrome associated with functional decline, dependency, and adverse health outcomes in older adults. Community-based nursing interventions may help prevent or delay frailty by promoting physical activity, health education, and self-care. This exploratory study aimed to describe pre–post changes in frailty status and functional capacity following a community-based nursing intervention among community-dwelling older women. **Methods:** An exploratory uncontrolled pre–post study was conducted in Spain. Of 35 community-dwelling, non-institutionalized women aged ≥65 years who underwent baseline assessment, 23 provided complete baseline and post-intervention data and were included in the final pre–post analysis. The intervention consisted of a 16-week nursing programme with weekly group sessions focused on adapted physical exercise, health education, and self-care. Frailty was assessed using the FRAIL Scale. Four available components of the Fried phenotype were examined as secondary frailty-related measures. Functional capacity was evaluated using the Barthel Index and the Lawton and Brody Scale. **Results:** Among the 23 participants included in the final paired pre–post analysis, the proportion classified as frail according to the FRAIL Scale decreased from 47.8% at baseline to 8.7% at post-intervention assessment, while the proportion classified as robust increased from 17.4% to 30.4%. Nine of the eleven participants classified as frail at baseline moved to a less severe frailty category at follow-up. Frailty status changed significantly across the three FRAIL categories (*p* < 0.001). Additional analyses showed a decrease in difficulty walking 100 m (43.5% to 8.7%; *p* = 0.008) and in low physical activity (60.9% to 26.1%; *p* = 0.021). Functional capacity did not change significantly. Participants reported high levels of satisfaction with the programme. **Conclusions:** Favourable pre–post changes in FRAIL status and selected frailty-related measures were observed among participants who completed the study, while functional capacity remained stable. Given the exploratory uncontrolled design, small analytical sample, and participant attrition, these findings should not be interpreted as evidence of intervention effectiveness. Larger controlled studies are required to determine whether the observed changes can be attributed to the intervention.

## 1. Introduction

Population ageing is one of the greatest public health challenges of the 21st century. Increased life expectancy and declining fertility rates have led to a substantial rise in the number of older adults worldwide, resulting in a higher prevalence of chronic diseases, disability, dependency, and increasing healthcare demands. Promoting healthy ageing has therefore become a priority for healthcare systems and policymakers worldwide [1,2,3,4].

Frailty is one of the most important geriatric syndromes associated with ageing. It is characterised by reduced physiological reserve and increased vulnerability to stressors and is associated with a higher risk of falls, disability, hospitalisation, institutionalisation, and mortality [5,6,7,8]. Unlike disability or multimorbidity, frailty is considered a dynamic and potentially reversible condition, making early identification and intervention particularly relevant for preserving functional capacity and independence in older adults [5,6,7]. Frailty is particularly relevant among older women, who consistently show a higher prevalence than men, highlighting the importance of preventive strategies in this population [7,9].

Regular physical exercise is one of the main strategies for preventing and managing frailty. Programmes combining strength, balance, mobility, and endurance training have been associated with improvements in physical performance, muscle function, and frailty status [10,11,12,13]. Systematic reviews and meta-analyses also support the role of exercise-based interventions in maintaining functional capacity and addressing frailty among community-dwelling older adults [11,12,13].

Community nursing also has an important role in frailty prevention and healthy ageing. Through early frailty screening, health education, self-care promotion, and regular follow-up, nurses can support healthy lifestyles, physical activity, and the maintenance of functional independence, particularly in primary care and community settings [14,15,16]. These activities are consistent with the preventive and health-promotion role of community nursing and provide opportunities for early identification and management of frailty [14,15,16].

Frailty is closely related to functional capacity, defined as the ability to perform basic and instrumental activities of daily living independently. Functional decline is one of the main adverse consequences associated with frailty and is frequently considered when evaluating changes in older adults over time. Several instruments are available to assess frailty. The FRAIL Scale is a brief five-item screening tool assessing Fatigue, Resistance, Ambulation, Illnesses, and Loss of Weight [8]. The Fried phenotype is another widely used approach based on five physical criteria: unintentional weight loss, exhaustion, weakness, slow walking speed, and low physical activity [6,7].

Although the benefits of exercise for frailty are well established, evidence on changes in frailty status and functional capacity following nursing-led community programmes remains limited, particularly among community-dwelling older women. Exploratory studies conducted in community settings can provide preliminary information on changes observed following these programmes and inform the design of subsequent controlled studies.

The aim of this exploratory study was to describe pre–post changes in frailty status and functional capacity following a 16-week community-based nursing intervention among community-dwelling older women.

## 2. Materials and Methods

### 2.1. Study Design

An exploratory, uncontrolled, longitudinal pre–post pilot study was conducted to describe changes in frailty status and functional capacity following a community-based nursing intervention among community-dwelling older women. The study was conducted under routine community conditions, with assessments performed before and after the 16-week intervention. The study was reported in accordance with the Transparent Reporting of Evaluations with Non-randomized Designs (TREND) statement for non-randomized intervention studies [17].

### 2.2. Setting and Participants

The study was conducted in a community setting in Bermeo (Spain) between December 2025 and April 2026. The target population consisted of community-dwelling women aged 65 years and older.

Participants were recruited through community advertisements and an information session where the objectives, procedures, and study timeline were explained. Convenience sampling was used, and no a priori sample size calculation was performed because recruitment included all eligible women who agreed to participate during the study period.

A total of 35 women underwent the initial screening and baseline assessment. Of these, 10 did not complete the programme because of insufficient attendance, and two did not complete the post-intervention assessment. The remaining 23 participants provided complete baseline and post-intervention data and were included in the final paired pre–post analysis.

### 2.3. Inclusion and Exclusion Criteria

Women were eligible to participate if they met the following inclusion criteria: (1) aged 65 years or older; (2) living in the community; (3) able to participate in group activities and adapted physical exercise sessions; and (4) willing to provide written informed consent.

Participants were excluded if they had cognitive impairment or physical limitations that prevented safe participation in the intervention.

### 2.4. Intervention

The intervention consisted of a 16-week community-based nursing programme designed and delivered by two registered nurses with experience in community and geriatric care. One nurse was responsible for coordinating and conducting the intervention, while the second participated in the design, planning, and supervision of the programme to ensure methodological consistency.

Before the intervention, all participants underwent an individual baseline assessment, including frailty screening, functional assessment, and a semi-structured interview exploring daily routines, lifestyle habits, perceived health status, functional limitations, and individual concerns. The information obtained during this assessment was used to adapt the educational content and practical activities to the characteristics, functional abilities, and specific needs of the group.

The programme was delivered over 16 consecutive weeks through weekly face-to-face group sessions held in a community setting. Sessions lasted between 60 and 120 min depending on the planned activities and participants’ progression. Attendance was recorded at each face-to-face session.

The intervention was based on three complementary components: adapted physical exercise, health education, and self-care promotion.

The physical exercise component focused on balance, mobility, muscle strength, coordination, and postural control, together with fall prevention. Exercise sessions progressed gradually throughout the programme. Initial sessions included low-intensity activities focused on joint mobility, gait, and balance. As participants progressed, resistance and functional exercises were incorporated to improve lower-limb strength and functional performance. Participants performed two sets of 5–8 repetitions of each exercise during the supervised sessions, and the same exercises were recommended for home practice between sessions. Exercise intensity and difficulty were individually adapted according to each participant’s physical condition, functional capacity, and tolerance. Illustrated examples of the exercises performed during the supervised sessions are provided in the Supplementary Materials to facilitate replication of the intervention.

The educational component addressed healthy ageing, frailty prevention, healthy lifestyles, physical activity, nutrition, medication adherence, fall prevention, environmental safety, and strategies to maintain autonomy. Educational sessions encouraged participants to recognise modifiable risk factors associated with functional decline and to incorporate healthy behaviours into their daily routines.

Self-care promotion was integrated throughout the intervention by encouraging participants to actively participate in their own health management. Practical recommendations were provided to facilitate healthy habits, confidence in daily activities, and an active lifestyle beyond the supervised sessions.

Group interaction was incorporated into the programme to facilitate peer support and social interaction. Participants were encouraged to share experiences, discuss everyday challenges, and exchange practical strategies related to ageing and maintaining independence.

To maintain continuity between sessions, participants received regular telephone follow-up and were invited to join a WhatsApp group. These strategies were used to provide reminders, support home-based exercise practice, facilitate communication with the research team, and maintain contact throughout the intervention period. Participants could also provide feedback to the nurse leading the intervention regarding the recommended home-based exercises through WhatsApp-based communication and telephone follow-up.

Participants were encouraged to perform the recommended exercises at home between sessions and received printed educational materials to support independent practice.

### 2.5. Outcome Measures

#### 2.5.1. Frailty Assessment

Frailty was assessed using the FRAIL Scale, a five-item screening instrument comprising Fatigue, Resistance, Ambulation, Illnesses, and Loss of Weight [8]. Each item was scored dichotomously (0 = criterion absent; 1 = criterion present), yielding a total score ranging from 0 to 5. Participants were classified as robust (0), pre-frail (1–2), or frail (3–5) [8].

The five FRAIL domains were assessed using the following items: frequent fatigue (Fatigue), difficulty climbing stairs (Resistance), difficulty walking 100 m (Ambulation), presence of five or more diagnosed chronic illnesses (Illnesses), and loss of more than 5% of body weight during the previous year (Loss of Weight). Individual FRAIL items were also analysed separately to describe changes in each domain.

In addition to the FRAIL Scale, four available components of the Fried frailty phenotype were examined as secondary frailty-related measures: unintentional weight loss, muscle weakness, slow gait speed, and low physical activity [6,7]. The exhaustion component was not available; therefore, a global Fried phenotype classification was not calculated.

Unintentional weight loss was assessed by participant self-report and was considered present when participants reported a significant involuntary change in body weight during the previous year.

Muscle weakness was assessed using handgrip strength measured with a handheld dynamometer. Grip strength was evaluated using sex- and body mass index-specific cut-off values. For women, the cut-off values used in the screening protocol were ≤17 kg, ≤17.3 kg, and ≤18 kg for the low, medium, and high BMI categories, respectively.

Slow gait speed was assessed using a timed 4.5-m walking test. Walking speed was calculated in metres per second, and a value <0.8 m/s was considered indicative of slow gait speed.

Low physical activity was assessed by participant-reported reduction in physical activity compared with the previous year, considering the activities performed approximately one year earlier and perceived changes since then.

All assessments were performed at baseline and post-intervention using the same protocol.

#### 2.5.2. Functional Capacity Assessment

Functional capacity was assessed using two validated instruments commonly applied in geriatric research.

The Barthel Index was used to evaluate independence in basic activities of daily living (ADL), including feeding, bathing, dressing, toileting, transfers, and mobility. Scores range from 0 to 100, with higher scores indicating greater functional independence [18].

Instrumental activities of daily living (IADL) were assessed using the Lawton and Brody Scale, which evaluates more complex activities required for independent community living. Higher scores indicate greater functional autonomy [19].

#### 2.5.3. Satisfaction Assessment

Participant satisfaction was evaluated descriptively at the end of the intervention using a four-point Likert-type questionnaire specifically developed for the programme. The questionnaire explored participants’ perceptions regarding the usefulness and quality of the intervention, motivation, and overall satisfaction, as well as their willingness to participate in similar programmes in the future. The questionnaire was developed specifically for this programme and was used for descriptive purposes only. The complete questionnaire, including all items, response options, scoring procedure, and criteria used to classify satisfaction levels, is provided in the Supplementary Materials.

### 2.6. Data Analysis

Descriptive statistics were used to summarise participants’ sociodemographic and clinical characteristics. Continuous variables are presented as means and standard deviations (SD), whereas categorical variables are expressed as frequencies and percentages.

The primary outcome was the change in overall frailty status between baseline and post-intervention assessment. Changes in individual frailty components, selected frailty-related indicators, and functional capacity were considered exploratory outcomes. Participant satisfaction was analysed descriptively at the end of the intervention.

Changes between baseline and post-intervention assessments were analysed using the McNemar test for dichotomous variables. Paired differences in proportions, expressed as percentage points (pp), and exact 95% confidence intervals (CI) were calculated to describe the magnitude of change. The exact marginal homogeneity test was used to assess changes in frailty status across the three FRAIL categories. As Barthel Index and Lawton and Brody Scale score changes were not normally distributed, differences between baseline and post-intervention were assessed using the Wilcoxon signed-rank test. Mean differences and 95% CIs were calculated using bootstrap resampling. Statistical significance was set at *p* < 0.05.

No adjustment for multiple comparisons was performed for the exploratory analyses. Accordingly, *p*-values for exploratory outcomes were interpreted as exploratory rather than confirmatory.

Statistical analyses were performed using IBM SPSS Statistics for Windows, Version 22.0 (IBM Corp., Armonk, NY, USA) [20] and the R environment [21]. Paired differences in proportions with confidence intervals were obtained using the *exact2x2* package version 1.7.0 [22]. The alluvial plot was generated using the *ggalluvial* package version 0.12.5 [23].

### 2.7. Ethical Considerations

The study was conducted in accordance with the ethical principles of the Declaration of Helsinki and complied with current Spanish legislation on biomedical research and personal data protection.

Ethical approval was obtained from the Bioethics and Biosafety Committee of the University of Extremadura (Reference No. 338/2025) before participant recruitment.

All participants received written and verbal information about the study and provided written informed consent prior to enrolment. Participation was voluntary, and participants were free to withdraw from the study at any time without consequences.

Participant confidentiality and anonymity were guaranteed throughout the study. Personal information was processed in accordance with Organic Law 3/2018 on Personal Data Protection and Guarantee of Digital Rights, and all data were analysed anonymously.

## 3. Results

### 3.1. Participant Characteristics

**Figure 1:**
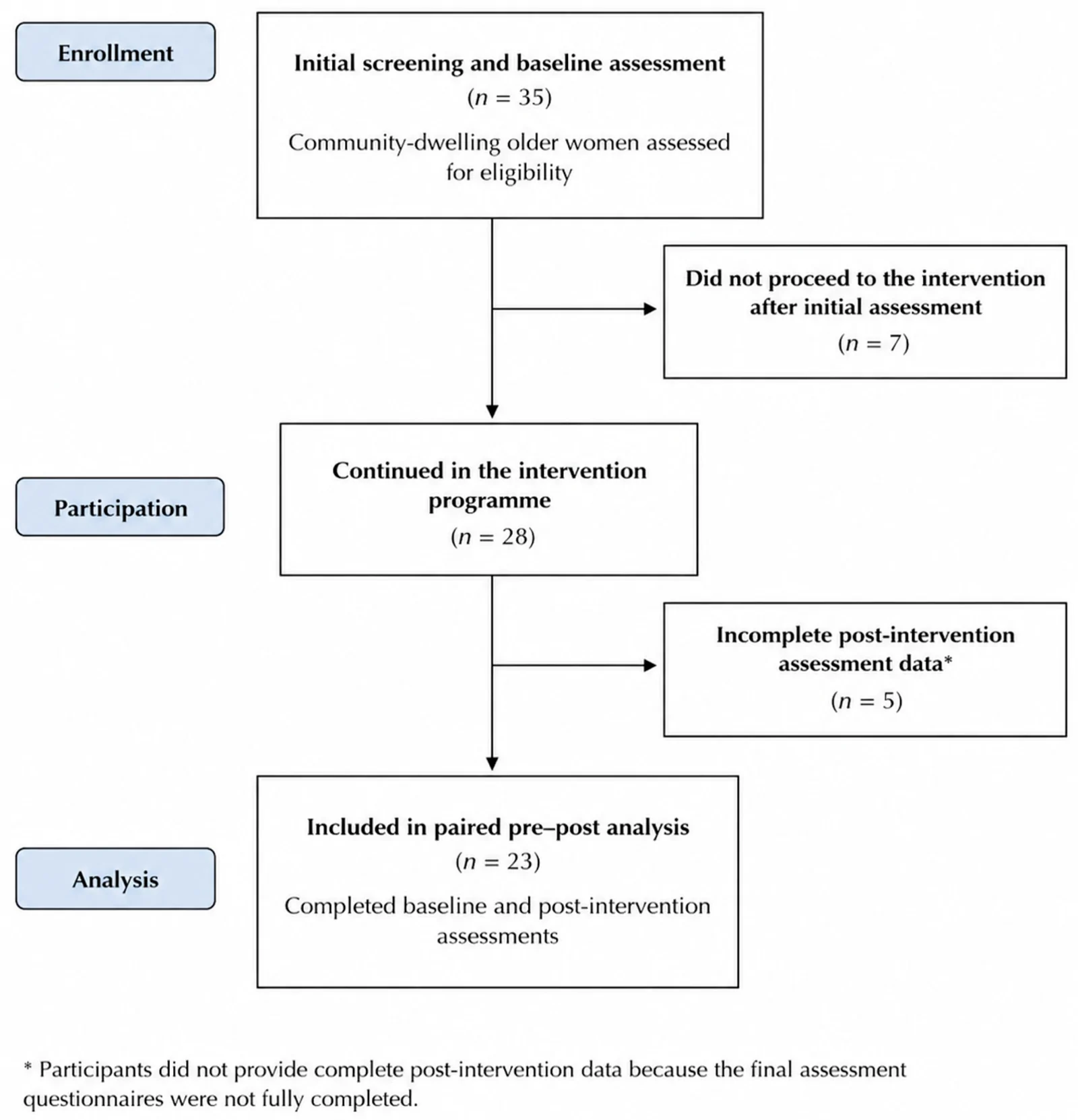
Participant flow throughout the study.

A total of 35 community-dwelling older women underwent the initial screening and baseline assessment. Seven did not proceed to the intervention after this initial stage. Of the 28 participants who continued in the programme, 23 provided complete baseline and post-intervention data and were included in the final paired pre–post analysis. Five participants did not provide complete post-intervention data because the final assessment questionnaires were not fully completed. The specific reasons for questionnaire non-completion were not systematically recorded. Participant flow throughout the study is shown in Figure 1.

The mean age of the 23 participants included in the final analysis was 77.6 ± 4.2 years. More than half lived alone (56.5%), whereas 43.5% lived with family members. Most participants had at least one chronic condition (82.6%), with hypertension being the most prevalent comorbidity, followed by osteoarthritis and diabetes mellitus. A complete comparison of baseline characteristics by follow-up status could not be performed because baseline information was incomplete for participants not included in the paired analysis. No missing outcome data were observed among the 23 participants included in the paired analysis. Baseline characteristics of the final analytical sample are presented in Table 1.

### 3.2. Changes in Frailty Status

Changes in frailty status between baseline and post-intervention assessment are presented in Table 2A. At baseline, 47.8% of participants were classified as frail, 34.8% as pre-frail, and 17.4% as robust. At the post-intervention assessment, these proportions were 8.7%, 60.9%, and 30.4%, respectively. The overall distribution of frailty status differed between baseline and post-intervention assessment (*p* < 0.001).

**Figure 2:**
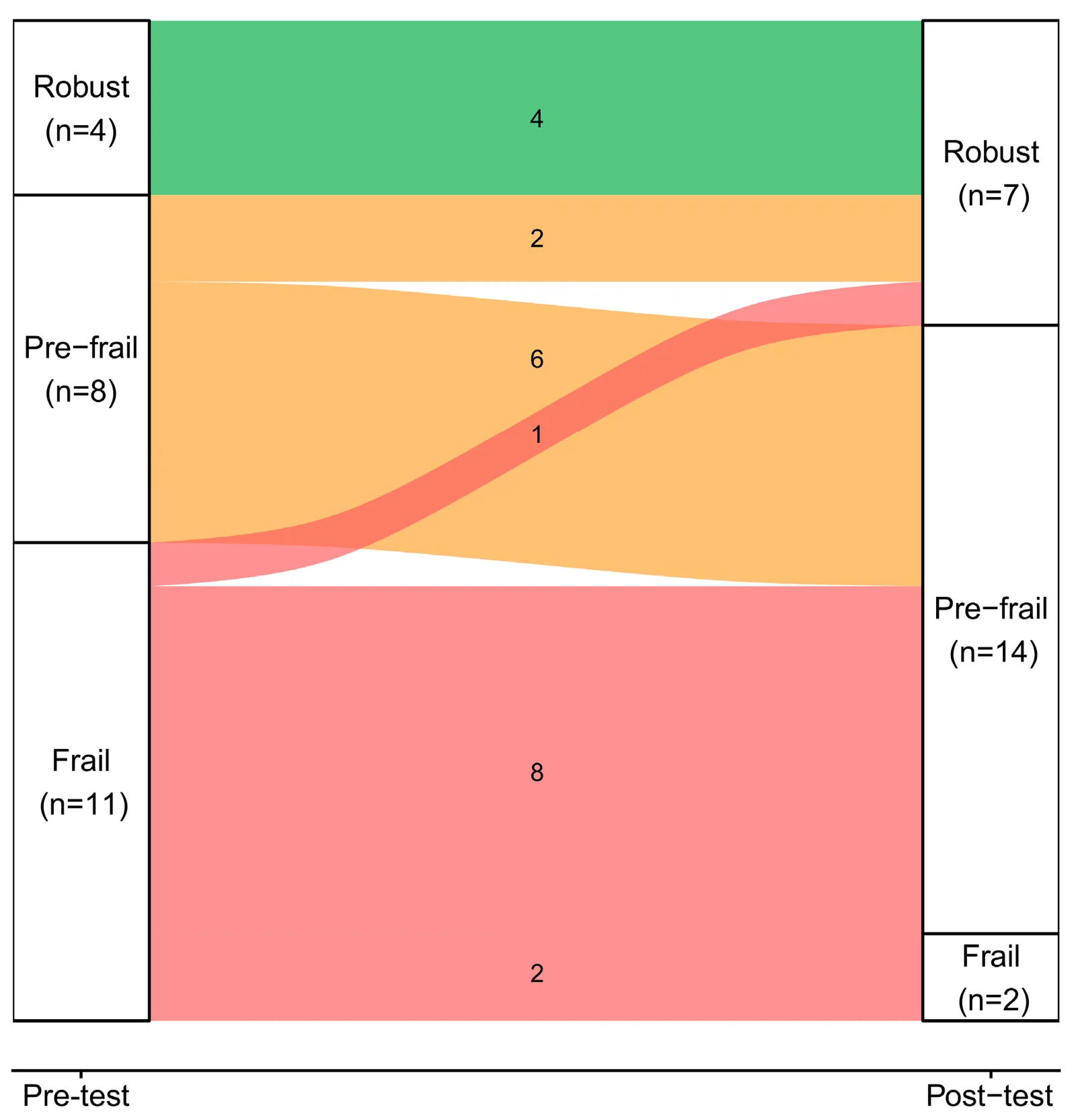
Individual transitions in FRAIL status from baseline to post-intervention assessment (n = 23). Colours indicate baseline FRAIL status: green for robust, orange for pre-frail, and red for frail. Numbers within the flows indicate the number of participants in each transition.

Analysis of transitions between frailty categories (Figure 2) showed that, of the eleven participants classified as frail at baseline, eight were classified as pre-frail and one as robust at the post-intervention assessment, while two remained frail. All four participants classified as robust at baseline maintained this status at the post-intervention assessment. In addition, two participants who were classified as pre-frail at baseline were classified as robust at follow-up.

Exploratory analyses of the individual FRAIL scale components showed a decrease in the proportion of participants reporting difficulty walking 100 m, from 43.5% at baseline to 8.7% at post-intervention assessment (*p* = 0.008). Among the available Fried phenotype components, low physical activity decreased from 60.9% to 26.1% (*p* = 0.021), while no statistically significant differences were observed in unintentional weight loss, muscle weakness, or slow gait speed. These secondary analyses were exploratory and were not adjusted for multiple comparisons. Complete results are presented in Table 2A,B.

### 3.3. Functional Capacity and Participant Satisfaction

Functional capacity remained relatively stable between baseline and post-intervention assessment. The mean Barthel Index score was 91.5 at baseline and 92.6 post-intervention, with no statistically significant change (*p* = 0.301). According to the Barthel Index functional status, 34.8% of participants were classified as completely independent at baseline compared with 39.1% at the post-intervention assessment, whereas mild dependence was observed in 65.2% and 60.9%, respectively. One participant moved from mild dependence to complete independence. No statistically significant difference was observed in Barthel Index functional status (*p* = 1.000).

Similarly, independence in instrumental activities of daily living remained largely unchanged between assessments. The mean Lawton and Brody Scale score was 7.9 at both baseline and post-intervention (*p* = 1.000). According to the Lawton and Brody Scale, 87.0% of participants were independent at baseline compared with 91.3% at the post-intervention assessment, whereas mild dependence was observed in 13.0% and 8.7%, respectively. No statistically significant difference was observed in Lawton and Brody functional status (*p* = 1.000).

At the end of the programme, 95.7% of participants reported a high level of satisfaction and 4.3% reported moderate satisfaction. In addition, 87.0% indicated that they would participate in a similar community-based programme in the future. Satisfaction outcomes were analysed descriptively. Functional capacity and participant satisfaction results are presented in Table 3.

## 4. Discussion

### 4.1. Principal Findings

This exploratory uncontrolled pre–post pilot study examined changes in frailty status and functional capacity following a 16-week community-based nursing programme among community-dwelling older women. The main finding was a change in the distribution of frailty status between baseline and post-intervention assessment. According to the FRAIL Scale, the proportion of participants classified as frail decreased from 47.8% to 8.7%, while the proportion classified as robust increased from 17.4% to 30.4%. Nine of the eleven participants classified as frail at baseline moved to a less severe frailty category at follow-up. Among the individual FRAIL items, the proportion reporting difficulty walking 100 m decreased, and among the four available Fried phenotype components, the proportion with low physical activity decreased from baseline to post-intervention assessment. Functional capacity, assessed using the Barthel Index and the Lawton and Brody Scale, remained largely unchanged.

The limited change in functional capacity should be considered in relation to the high level of independence already observed at baseline. Most participants presented little or no functional dependence, leaving limited scope for substantial change over the 16-week period. Preservation of functional capacity is nevertheless relevant in older populations, although the uncontrolled design does not allow this finding to be attributed to the programme.

Participant satisfaction was high among women who completed the post-intervention assessment, with 95.7% reporting high satisfaction and 87.0% indicating that they would participate in a similar programme in the future. These results provide descriptive information about participants’ perceptions of the programme but should not be interpreted as formal evidence of acceptability or feasibility, as these constructs were not assessed using validated measures.

The programme combined supervised physical exercise with health education, self-care promotion, fall prevention strategies, environmental recommendations, group participation, and regular follow-up. This multicomponent approach is consistent with current evidence supporting exercise and multidimensional strategies for frailty management in community-dwelling older adults [11,13,14,15].

### 4.2. Frailty as a Reversible Condition

Frailty is increasingly recognised as a dynamic clinical condition rather than an inevitable consequence of ageing. It is characterised by reduced physiological reserve and increased vulnerability to stressors, and transitions between different frailty states may occur over time [5,6,24]. This understanding reinforces the importance of early identification and management, particularly in community settings where many older adults remain functionally independent [16,24].

In the present study, according to the FRAIL Scale, the proportion of participants classified as frail decreased from 47.8% at baseline to 8.7% at the post-intervention assessment, while the proportion classified as robust increased from 17.4% to 30.4%. Of the eleven women classified as frail at baseline, eight were classified as pre-frail and one as robust at the post-intervention assessment, while two remained frail. These findings describe transitions between frailty categories during the study period. Given the uncontrolled design, it is not possible to determine whether these changes were related to the intervention or to other factors.

The clinical relevance of frailty lies in its association with adverse health outcomes and its potential progression towards disability, dependence, institutionalisation, and mortality [24,25,26]. Identifying changes in frailty status before substantial functional deterioration occurs is therefore particularly relevant in community-dwelling older adults. In this study, changes in frailty classification were observed while basic and instrumental functional capacity remained largely unchanged.

This perspective is consistent with current international strategies for healthy ageing. The WHO Decade of Healthy Ageing 2021–2030 emphasises maintaining functional ability and creating opportunities for older people to preserve their health and participation as they age [2]. Keating et al. also highlight the importance of approaches that consider individual capacity together with the environments in which older adults live [3]. The present findings provide preliminary information to support further investigation of community-based programmes using controlled designs and larger samples.

### 4.3. The Contribution of Community Nursing to Frailty Management

Physical exercise is a central component of frailty management. Interventions incorporating strength, balance, mobility, and aerobic exercise have been associated with improvements in physical performance and frailty-related outcomes among frail and pre-frail older adults [10,11,13]. International clinical practice guidelines also recognise physical activity and exercise as important components of frailty management [27].

The programme examined in this study was developed from a nursing perspective and extended beyond supervised physical exercise. Participants received education on healthy ageing, self-care, fall prevention, environmental adaptation, and strategies for incorporating physical activity into their daily routines. Telephone follow-up and group communication were also used to maintain contact between face-to-face sessions. These components are consistent with the preventive, educational, and follow-up functions of community nursing and with approaches aimed at the early identification and management of frailty [14,15,16].

The changes observed in some frailty outcomes are consistent with previous evidence on exercise-based interventions. Yang et al. reported favourable effects of multicomponent exercise on frailty status and physical function in frail older adults [11]. Luo et al. also found benefits of multicomponent exercise among older adults with cognitive frailty [12]. Similarly, Racey et al. reported improvements in frailty-related outcomes following physical activity interventions among frail and pre-frail older adults [13]. These findings support the inclusion of structured physical activity within community strategies addressing frailty.

The nursing component is also relevant. Kasa et al. examined nurse-led interventions for the management of frailty in community-dwelling older people and identified potential benefits of approaches incorporating assessment, education, support, and follow-up [14]. Zheng et al. similarly reported favourable physical and mental health outcomes associated with nurse-led interventions among frail and pre-frail older adults [15]. Community nurses are therefore well positioned to identify frailty, provide health education, adapt recommendations to individual needs, and support continuity of care.

The present study cannot determine which component of the programme, if any, contributed to the observed changes. Its multicomponent nature prevents the independent contribution of physical exercise, health education, self-care promotion, group interaction, or follow-up from being established. The results should therefore be interpreted as changes observed following the programme as a whole rather than effects attributable to any individual component.

The 16-week duration allowed progressive implementation of the exercise and educational components and continued contact with participants. Current recommendations emphasise regular physical activity and sustained strategies for the management of frailty and maintenance of functional capacity [26,28]. Future controlled studies should examine the duration, intensity, and combination of components required to achieve clinically meaningful and sustained changes.

### 4.4. Limitations

This study has several limitations. The uncontrolled design, small analytical sample (n = 23), and convenience sampling limit causal interpretation and generalisability. Although no marked baseline differences were apparent between participants with complete and incomplete post-intervention data based on the available information, attrition bias cannot be excluded. Secondary outcomes were explored without adjustment for multiple comparisons and should therefore be interpreted cautiously. Satisfaction was assessed using a non-validated programme-specific questionnaire, and only women were included. Larger controlled studies are needed to confirm these findings.

## 5. Conclusions

This exploratory uncontrolled pre–post pilot study identified changes in frailty status among community-dwelling older women following a 16-week community-based nursing programme. The proportion of participants classified as frail decreased, while the proportion classified as robust increased. Exploratory analyses also showed changes in fatigue and low physical activity, whereas functional capacity remained largely unchanged between baseline and post-intervention assessment.

These findings should be interpreted as preliminary pre–post changes rather than evidence of intervention effectiveness. Further controlled studies with larger samples are needed to determine whether community-based nursing programmes are associated with changes in frailty status and functional capacity among older adults.

## Figures and Tables

**Table 1:** Baseline characteristics of participants included in the final paired analysis (n = 23).

Variable	Value
Age, mean (SD)	77.6 (4.2)
Living alone	13 (56.5%)
Living with family	10 (43.5%)
Professional support	4 (17.4%)
Comorbidities	19 (82.6%)
Hypertension	15 (65.2%)
Osteoarthritis	8 (34.8%)
Diabetes mellitus	7 (30.4%)
Joint injury/sequelae	7 (30.4%)

**Table 2:** (**A**) Changes in frailty status according to the FRAIL scale and its individual components between baseline and post-intervention assessment (n = 23). (**B**) Changes in available Fried phenotype components between baseline and post-intervention assessment (n = 23).

(**A**)				
**Variable**	**Pre-Test n (%)**	**Post-Test n (%)**	**Change pp (95% CI)**	***p*** **-Value**
**Frailty status**				***<0.001***
Robust	4 (17.4)	7 (30.4)	13.0 (−6.8, 33.6)	
Pre-frail	8 (34.8)	14 (60.9)	26.1 (−5.0, 52.7)	
Frail	11 (47.8)	2 (8.7)	−39.1 (−61.5, −10.2)	
**Individual Frail Scale components**				
Fatigue (frequent fatigue)	14 (60.9)	13 (56.5)	−4.3 (−25.5, 16.6)	*1.000*
Resistance (difficulty climbing stairs)	14 (60.9)	9 (39.1)	−21.7 (−46.0, 5.0)	*0.125*
Ambulation (difficulty walking 100 m)	10 (43.5)	2 (8.7)	−34.8 (−57.3, −7.2)	***0.008***
Illnesses (having ≥5 diagnosed chronic illnesses)	1 (4.3)	2 (8.7)	4.3 (−12.2, 21.9)	*1.000*
Loss of weight (weight loss >5% during the previous year)	5 (21.7)	1 (4.3)	−17.4 (−41.3, 8.1)	*0.219*
(**B**)				
**Variable**	**Pre-Test n (%)**	**Post-Test n (%)**	**Change pp (95% CI)**	***p*** **-Value**
Unintentional weight loss	1 (4.3)	0 (0.0)	−4.3 (−21.9, 12.2)	*1.000*
Muscle weakness	16 (69.6)	12 (52.2)	−17.4 (−43.6, 11.3)	*0.289*
Slow gait speed	5 (21.7)	7 (30.4)	8.7 (−17.2, 33.7)	*0.688*
Low physical activity	14 (60.9)	6 (26.1)	−34.8 (−59.3, −4.2)	***0.021***

Note: bold indicates statistically significant *p*-values (*p* < 0.05).

**Table 3:** Functional capacity at baseline and post-intervention assessment and participant satisfaction at the end of the programme (n = 23).

Outcome	Pre-Test	Post-Test	Change, Mean Difference or pp (95% CI)	*p*-Value
**Barthel Index score**	91.5 (9.7)	92.6 (8.0)	1.1 (−0.7, 3.0)	*0.301*
**Barthel Index functional status**				*1.000*
Complete independence	8 (34.8)	9 (39.1)		
Mild dependence	15 (65.2)	14 (60.9)	−4.3 (−25.5, 16.6)	
**Lawton and Brody Scale score**	7.9 (0.3)	7.9 (0.5)	0.0 (−0.2, 0.2)	*1.000*
**Lawton and Brody Scale functional status**				*1.000*
Independence	20 (87.0)	21 (91.3)		
Mild dependence	3 (13.0)	2 (8.7)	−4.3 (−25.5, 16.6)	
**Participant satisfaction**				
High satisfaction	—	22 (95.7)		*—*
Moderate satisfaction	—	1 (4.3)		*—*
Willingness to participate again	—	20 (87.0)		*—*

## Data Availability

The data presented in this study are available from the corresponding author upon reasonable request. The data are not publicly available due to privacy and ethical restrictions related to the protection of participants’ personal information.

## Supplementary Materials

The following supporting information can be downloaded at: https://www.mdpi.com/article/10.3390/healthcare14183060/s1, S1. Workshop Recruitment Poster. S2. Information Leaflet on Frailty and the Workshops. S3. Informed Consent Form. S4. Barthel Index. S5. Lawton and Brody Scale. S6. Frailty Assessment Form. S7. Exercise Programme. S8. Participant Satisfaction Questionnaire.

## Abbreviations

The following abbreviations are used in this manuscript:

ADL	Activities of Daily Living
IADL	Instrumental Activities of Daily Living
SD	Standard Deviation
