# Supplementary material for: Changes in Frailty Status and Functional Capacity Following a Community-Based Nursing-Led Intervention in Older Women: An Exploratory Uncontrolled Pre–Post Study
Source: Healthcare (Basel). 2026 Sep 17;14(18):3060. doi: 10.3390/healthcare14183060 (PMC13606484; doi:10.3390/healthcare14183060)
Supplement: Supplementary file 1 [file healthcare-14-03060-s001.zip › healthcare-4540768-supplementary.pdf]

SUPPLEMENTARY MATERIALS

Changes in Frailty Status and Functional Capacity Following a Community-Based Nursing-Led Intervention in Older Women: An Exploratory Uncontrolled Pre–Post Study

English version of study materials

Supplementary Material S1. Workshop Recruitment Poster

REGAIN YOUR VITALITY AND FEEL BETTER EVERY DAY!

Join a programme designed for your well-being.
100% FREE.

Do you notice tiredness, loss of strength, or instability?

You do not have to settle for it. We will show you how to improve.

WHAT WILL WE DO?

Through practical workshops, you will learn:

- Simple exercises to improve strength and balance.
- Simple habits that can make a big difference in everyday life.
- Tips to prevent frailty and falls.

WE OFFER SUPPORT AND KNOWLEDGE

You are not alone in this. Improving your well-being is important for you and those around you.

Supplementary Material S1. Workshop Recruitment Poster

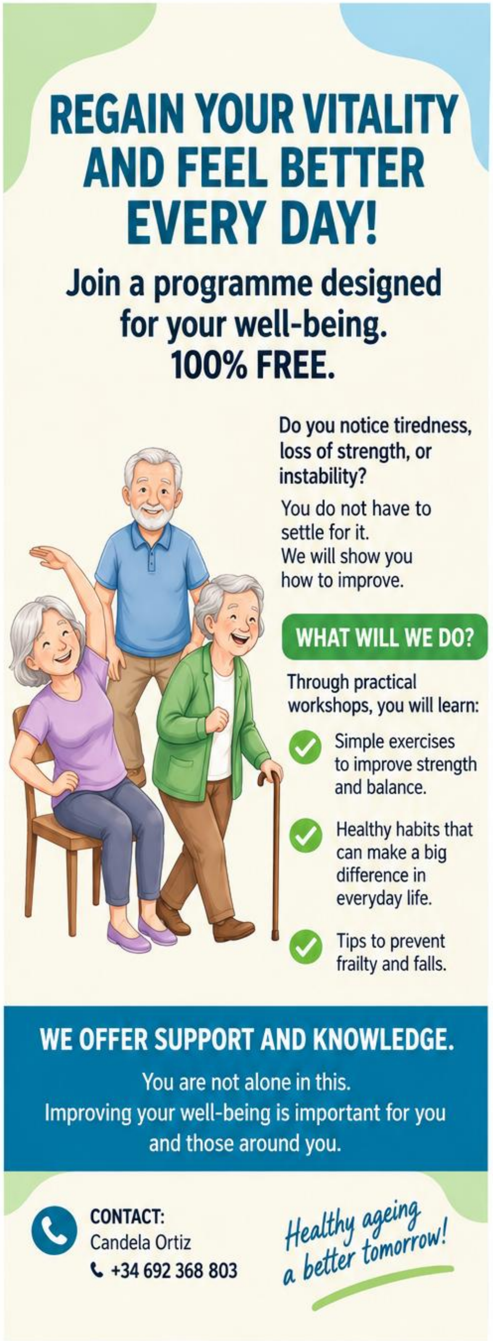

Supplementary Material S2. Information Leaflet on Frailty and the Workshops

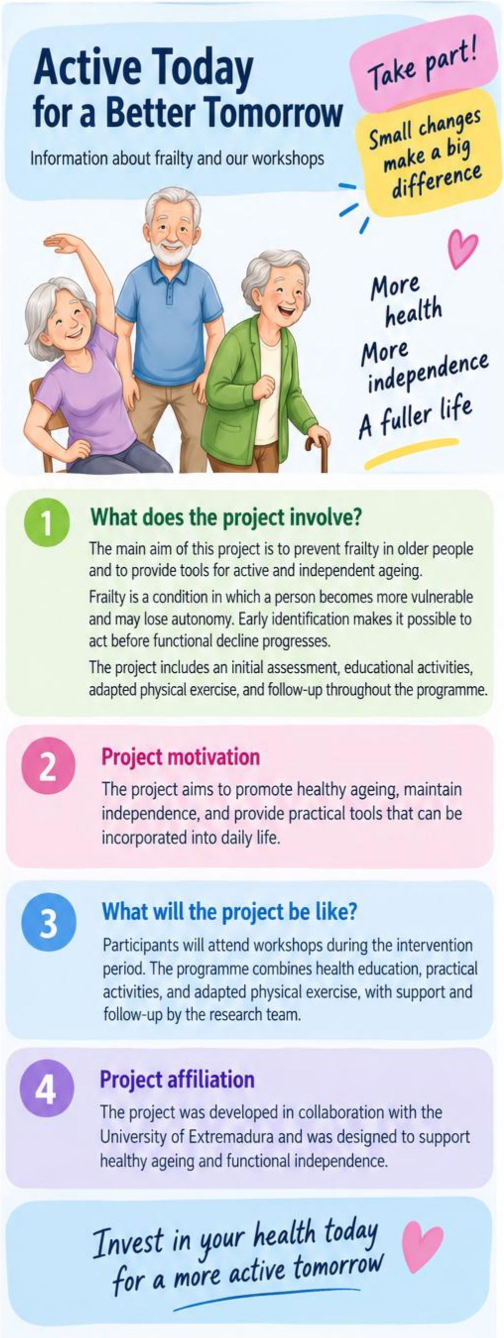

Supplementary Material S2. Information Leaflet on Frailty and the Workshops

What does the project involve?

The first activity of this project is to prevent frailty in older people and to provide tools for active and independent ageing.

Frailty is a condition in which a person becomes more vulnerable and may lose autonomy. Early identification makes it possible to act before functional decline progresses.

The project includes an initial assessment, educational activities, adapted physical exercise, and follow-up throughout the programme.

Project motivation

The project aims to promote healthy ageing, maintain independence, and provide practical tools that can be incorporated into daily life.

What will the project be like?

Participants will attend workshops during the intervention period. The programme combines health education, practical activities, and adapted physical exercise, with support and follow-up by the research team.

Project affiliation

The project was developed in collaboration with the University of Extremadura and was designed to support healthy ageing and functional independence.

Supplementary Material S3. Informed Consent Form

INFORMED CONSENT

Project: Nursing intervention for the detection and management of frailty in older adults: a pre-post study with follow-up.

Participant: ____

Researcher(s): ______________________________________

The principal investigator may be contacted at any time to request information about the project through the contact details provided by the research team.

PLEASE READ THE INFORMATION CONTAINED IN THIS DOCUMENT CAREFULLY AND MAKE SURE THAT YOU UNDERSTAND THIS RESEARCH PROJECT. IF YOU AGREE TO PARTICIPATE IN THIS STUDY, PLEASE SIGN THIS DOCUMENT. BY SIGNING, YOU ACKNOWLEDGE THAT YOU HAVE BEEN INFORMED ABOUT THE CHARACTERISTICS, REQUIREMENTS, AND RISKS OF THE PROJECT AND THAT YOU FREELY AGREE TO PARTICIPATE. YOU WILL RECEIVE A COPY OF THIS DOCUMENT.

PURPOSE OF THE STUDY

You have been invited to participate in a research study aimed at developing a method for the early detection of frailty in older adults, together with an educational and self-care intervention intended to prevent or reverse this condition.

This project seeks to improve the ageing process by increasing participants' knowledge and autonomy and thereby supporting quality of life during this stage of life.

STUDY PROCEDURES AND DURATION

You will be asked to complete physical tests, including handgrip strength measurement using a dynamometer and a timed 4.5-m walking test, as well as questionnaires at the beginning and end of the project to assess physical and mental status before and after the intervention.

You will participate in face-to-face educational workshops of approximately one hour, including talks related to frailty as well as individual and group discussions to explore the project topics in greater depth.

The project will last approximately five months. The intervention will be conducted solely for research purposes and without commercial profit.

STUDY RESULTS

At the end of the study, you may be informed of the overall study results if you wish, but not of your individual results. All information will be treated confidentially in accordance with the Declaration of Helsinki and Spanish Law 14/2007 on Biomedical Research.

RISKS ARISING FROM PARTICIPATION

This study does not involve anticipated physical or psychological risks. The workshops will be conducted in safe settings.

BENEFITS

Your participation will contribute to improving care for older people with frailty by facilitating early detection and follow-up. Participants may also benefit from direct attention and participation in the educational workshops.

COSTS

Participation will not involve any financial cost to the participant. All materials and resources will be covered by the project.

CONFIDENTIALITY OF YOUR DATA

In accordance with current legislation, all information obtained will be treated confidentially. Data-collection records will be stored securely, and each participant will be assigned a code so that the information obtained cannot be directly linked to the participant's identity. Data will be anonymised for analysis and possible subsequent analysis.

The principal investigator undertakes to ensure strict confidentiality of the data obtained in this project. Personal data will be known only to the project researchers. Where appropriate, participants will be informed if any project result is considered potentially relevant to them.

The principal investigator undertakes not to use samples/data for studies other than this project and not to transfer samples/data to other projects or research teams.

PERSONAL DATA PROTECTION

For matters not covered in this document, current Spanish legislation on personal data protection (Organic Law 3/2018 of 5 December on Personal Data Protection and guarantee of digital rights), biomedical research (Law 14/2007 of 3 July on Biomedical Research), and any other applicable legislation shall apply.

Study results may be published in scientific journals or general publications. Information concerning your participation will remain confidential.

You will receive a copy of this Informed Consent Form signed by you.

PARTICIPANT DECLARATION

- The purpose and characteristics of the project.
- The advantages and disadvantages of this procedure.
- The purpose for which my data will be used.
- The fact that the data I provide will be used exclusively for non-profit research purposes.
- The fact that my data will be provided anonymously to the project researchers.
- The fact that I may request general information at any time about the studies for which my samples/data have been used.
- My right to withdraw consent at any time without repercussions.

I have understood the information received and have had the opportunity to ask all questions I considered appropriate.

You have the right to participate or not participate in the research and to withdraw your consent at any time. Choosing not to participate will not result in any disadvantage.

I HAVE BEEN PROVIDED WITH A COPY OF THIS DOCUMENT. I AGREE TO PARTICIPATE IN THIS STUDY.

Full name: ____

Signature: ____

Place and date: _______________________________________

Researcher's declaration that the participant has been duly informed.

Name: _______________________________ Signature: _______________________________

Supplementary Material S4. Barthel Index

Activity	Capacity	Score
Feeding	Fully independent	10
	Needs help cutting food, spreading butter, etc.	5
	Dependent	0
Bathing	Independent (or in shower)	5
	Dependent	0
Grooming	Independent: face/hair/teeth/shaving	5
	Dependent	0
Dressing	Independent, including buttons/zips/laces	10
	Needs help but can do about half unaided	5
	Dependent	0
Bowels	Continent	10
	Occasional accident	5
	Incontinent	0
Bladder	Continent	10
	Occasional accident	5
	Incontinent or catheterised and unable to manage alone	0
Toilet use	Independent	10
	Needs some help	5
	Dependent	0
Transfers	Independent	15
	Minor help	10
	Major help but able to sit unsupported	5
	Unable	0
Mobility	Independent for 50 m	15
	Walks 50 m with help	10
	Independent wheelchair use	5
	Immobile	0
Stairs	Independent	10
	Needs help or supervision	5
	Unable	0

Supplementary Material S5. Lawton and Brody Scale

Activity	Capacity	Score
Telephone	Uses telephone on own initiative; looks up and dials numbers	1
	Dials a few well-known numbers	1
	Answers telephone but does not dial	1
	Does not use telephone	0
Shopping	Takes care of all shopping needs independently	1
	Shops independently for small purchases	0
	Needs to be accompanied on any shopping trip	0
	Completely unable to shop	0
Food preparation	Plans, prepares, and serves adequate meals independently	1
	Prepares adequate meals if ingredients are supplied	0
	Heats and serves prepared meals but does not maintain adequate diet	0
	Needs meals prepared and served	0
Housekeeping	Maintains house alone or with occasional assistance for heavy work	1
	Performs light daily tasks such as dishwashing and bed making	1
	Performs light tasks but cannot maintain acceptable cleanliness independently	1
	Needs help with all home maintenance tasks	1
	Does not participate in housekeeping	0
Laundry	Does personal laundry completely	1
	Launders small items	1
	All laundry must be done by others	0
Transportation	Travels independently on public transport or drives own car	1
	Arranges own travel by taxi but does not use public transport	1
	Travels on public transport when accompanied	1
	Travels only by taxi or car with assistance	0
	Does not travel	0
Medication	Responsible for taking medication in correct dosages at correct time	1
	Takes medication if prepared in advance	0
	Unable to manage own medication	0
Finances	Manages financial matters independently	1
	Manages day-to-day purchases but needs help with banking or major purchases	1
	Unable to handle money	0

Supplementary Material S6. Frailty Assessment Form

A. FRAIL Scale

Domain	Question	Response
Fatigue	Do you frequently feel fatigued?	Yes / No
Resistance	Do you have difficulty climbing stairs?	Yes / No
Ambulation	Do you have difficulty walking 100 metres?	Yes / No
Illnesses	Do you have five or more chronic diseases?	Yes / No
Loss of weight	Have you lost more than 5% of your body weight during the previous year?	Yes / No

Each affirmative response is recorded as one point. Total FRAIL score: ____ / 5.

Classification: 0 = robust; 1-2 = pre-frail; 3-5 = frail.

B. Additional Fried phenotype components collected as exploratory secondary measures

1. Unintentional weight loss: Have you noticed a significant involuntary change in your body weight during the previous year? Criterion met: Yes / No

2. Muscle weakness: Handgrip strength measured with a dynamometer.

Sex	Low BMI	Medium BMI	High BMI
Women	≤17 kg	≤17.3 kg	≤18 kg
Men	≤29 kg	≤30 kg	≤32 kg

3. Slow gait speed: Time required to walk 4.5 m is recorded and walking speed is calculated. A cut-off value of <0.8 m/s is used.

Distance	Total time	m/s	Criterion met
4.5 m			Yes / No

4. Low physical activity:

- Have you noticed a decrease in your level of physical activity?
- What physical activities did you perform approximately one year ago?
- What changes have you noticed since then?

Note: A global Fried phenotype classification is not derived from this form because the validated Fried exhaustion criterion was not collected.

Supplementary Material S7. Exercise Programme

Side A. Strength and Resistance Exercises

Warm-up

No.	Exercise	Instructions
1	Ankle mobility	Standing, lift one foot and make circles with the ankle, first in one direction and then the other.
2	Knee mobility	Slightly flex one knee and then extend it, bringing the heel gently towards the buttock. Alternate legs.
3	Hip mobility	Standing with hands on the waist, make hip circles first in one direction and then the other.
4	Shoulder mobility	Standing, circle both shoulders forwards and backwards. Move gently and within a comfortable range.
5	Neck mobility	Move the head gently from side to side and then up and down, avoiding abrupt movements.

Strength and resistance exercises

No.	Exercise	Instructions
1	Shoulders	Holding the selected weight in both hands, start with the arms extended downwards and raise them laterally to shoulder height. Repeat 10 times.
2	Elbows	With the selected weight in both hands and arms extended, flex the elbows to raise and lower the weight. Repeat 10 times.
3	Wrist	Seated, rest the forearms on the thighs and move the wrists up and down. Perform 10 repetitions.
4	Sit-to-stand	With bottles held in the hands, rise from a chair and sit down while maintaining good posture. If needed, use the armrests for support. Repeat 10 times.
5	Shoulders without weights	With hands at waist level, move the arms forwards and backwards. Repeat 10 times.
6	Seated knee extension	Seated on a chair with the back straight, straighten one leg and then lower it. Alternate legs. Repeat 10 times.
7	Heel and toe raises	Standing and using a chair or wall for support, rise onto the toes and then lift the toes while keeping the heels on the floor.

Side B. Balance and Fall-Prevention Exercises

No.	Exercise	Instructions
1	Lateral trunk inclination	Standing in an anatomical position, lean the trunk to one side and then the other as far as possible while maintaining balance. Repeat 10 times.
2	Forward/backward trunk inclination	Standing in an anatomical position, lean the trunk forwards and backwards as far as possible while maintaining balance. Repeat 10 times.
3	Tandem stance	Stand with one foot directly in front of the other, shifting body weight towards the front foot. Repeat 10 times with each foot.
4	Lateral step	Repeat the previous movement towards one side and then the other. Repeat 10 times on each side.
5	Step exercise	Step up onto a step, hold for a few seconds, and step down. Repeat 10 times with each foot.
6	Forward step towards wall	Facing a wall, place the hands on the wall and step forwards. Repeat 10 times, approximately 10 steps.
7	Side stepping along wall	Using one hand on the wall for support, walk sideways while alternately moving the legs. Repeat 10 times in each direction.

Exercises were adapted to each participant's functional capacity and tolerance. During supervised sessions, participants performed two sets of 5-8 repetitions of each selected exercise, with progression according to individual capacity.

Supplementary Material S8. Participant Satisfaction Questionnaire

Circle the response that best represents your opinion for each question.

1 = lowest level / very dissatisfied; 4 = highest level / very satisfied.

Item	Question	Response options
1	How would you rate the quality of the workshops you received?	4 Excellent | 3 Good | 2 Fair | 1 Poor
2	Did you receive the type of information and tools you needed?	4 Definitely yes | 3 Generally yes | 2 In only a few cases | 1 No
3	Would you recommend these workshops to another person?	4 Definitely yes | 3 Probably yes | 2 Probably not | 1 Definitely not
4	How satisfied are you with the help provided by the workshops in addressing your problems?	4 Very satisfied | 3 Moderately satisfied | 2 Somewhat dissatisfied | 1 Very dissatisfied
5	Overall, how satisfied are you with the workshops?	4 Very satisfied | 3 Moderately satisfied | 2 Indifferent or slightly satisfied | 1 Not at all satisfied
6	During the workshops, my questions were adequately addressed.	4 Very satisfied | 3 Moderately satisfied | 2 Somewhat dissatisfied | 1 Very dissatisfied
7	How satisfied are you with the personal outcomes of the training received during the study?	4 Very satisfied | 3 Moderately satisfied | 2 Somewhat dissatisfied | 1 Very dissatisfied
8	How satisfied are you with the usefulness of what you learned in the workshops for everyday life?	4 Very satisfied | 3 Moderately satisfied | 2 Somewhat dissatisfied | 1 Very dissatisfied
9	How satisfied are you with the duration of the study and the workshops?	4 Very satisfied | 3 Moderately satisfied | 2 Somewhat dissatisfied | 1 Very dissatisfied
10	How motivated did you feel during the workshops?	4 Very motivated | 3 Moderately motivated | 2 Not very motivated | 1 Not at all motivated
11	I felt comfortable with the level of difficulty of the workshops.	4 Very comfortable | 3 Moderately comfortable | 2 Somewhat uncomfortable | 1 Very uncomfortable

What would you improve about the study?

____

If you could, would you continue participating in similar workshops?

____

RESULT: ______________________________

INTERPRETATION AS RECORDED ON THE ORIGINAL FORM:

10-20 → Low satisfaction

21-30 → Moderate satisfaction

31-44 → High satisfaction
